# Drug Monitoring of Therapy with Midazolam in Patients with ARDS: A Single-Center Prospective Study

**DOI:** 10.3390/medicina62040742

**Published:** 2026-04-13

**Authors:** Marek Grochla, Marcin Basiak, Bogusław Okopień, Piotr Knapik

**Affiliations:** 1 Department of Anesthesiology and Intensive Therapy in Zabrze, Medical University of Silesia in Katowice, 41-800 Zabrze, Poland; 2 Department of Internal Medicine and Clinical Pharmacology in Katowice, Medical University of Silesia in Katowice, 40-752 Katowice, Poland

**Keywords:** midazolam, drug monitoring, ARDS, ICU

## Abstract

*Background and Objectives*: One of the two primary classes of drugs administered in ICUs for pharmacological sedation is benzodiazepines. Among these, anesthesiologists consider midazolam the most commonly used and clinically significant agent. *Materials and Methods*: A prospective, single-center investigation involving 25 patients was carried out in the ICU. The study population consisted of patients undergoing mechanical ventilation with an FiO_2_ exceeding 60%, as well as ventilated individuals requiring additional support such as ECMO, NO, or ECCOR over 24 h before the study. Participants under 18 years of age or those not receiving continuous midazolam infusion were excluded. Measurements obtained from RASS and BIS were then compared with serum midazolam concentrations. On each day, when blood samples for midazolam measurements were taken, additional laboratory tests assessing renal and hepatic function were also carried out. *Results*: A negative correlation was shown between RASS and midazolam dosage (r = −0.44, *p* < 0.001), midazolam concentration (r = −0.33, *p* < 0.001), and α-OH-midazolam concentration (r = −0.24, *p* = 0.008). Similarly, a negative correlation was shown between BIS and midazolam concentration (r = −0.3, *p* = 0.016), as well as α-OH-midazolam (r = −0.3, *p* = 0.016). We observed that deceased patients received higher doses of midazolam to maintain the minimum level of required sedation compared to the others (135.5 ± 75.1 mg vs. 39.6 ± 59.2 mg; *p* = 0.002), indicating that these patients had higher concentrations of both midazolam and α-OH-midazolam (148.6 ± 83.5 µg/L vs. 27.2 ± 36.1 µg/L; *p* < 0.001, and 18 ± 15.9 vs. 5.3 ± 6.1 µg/L; *p* < 0.001). *Conclusions*: The results show that routine monitoring of midazolam does not provide additional clinical value. However, further studies are needed in high-risk groups. Despite the high mortality rate in the ICU for patients with severe respiratory failure, the six-month survival rate for discharged patients was high, exceeding 80%.

## 1. Introduction

Benzodiazepines constitute the primary class of agents used to sedate critically ill patients. Among these, midazolam is considered by anesthesiologists as the most commonly used and clinically significant agent. This agent acts as an agonist of gamma-aminobutyric acid (GABA) receptors [1,2,3].

Pharmacological interactions involving benzodiazepines are common and should be carefully monitored. These agents induce amnesia, which, in the context of ICU (Intensive Care Unit) treatment, is often considered beneficial, as it helps patients forget the typically distressing experiences associated with critical care [1,2,3].

However, prolonged ICU stays may lead to the development of tolerance, where increasing doses of the drug are required to achieve the same therapeutic effect. Additionally, the risk of benzodiazepine dependence necessitates a gradual tapering of midazolam, a process that may be lengthy but remains feasible in clinical practice [2,3,4,5].

ARDS (acute respiratory distress syndrome) patients are very difficult to treat in the ICU. Physicians require a biomarker to predict ARDS because once a patient presents hypoxemia, it is too late for further intervention. Potential ARDS biomarkers include RAGE (Receptor for Advanced Glycation Endproducts) and surfactant protein D. RAGE protein is a receptor protein present on the cells of the nervous system and epithelium, in addition to other locations. This protein binds AGEs (Advanced Glycation Endproducts), which are formed in aging cells. There are also reports that the RAGE protein is activated in ARDS or pneumonia [6]. Surfactant protein D is a mixture of phospholipids and proteins present in the lungs that reduce the surface tension between gases and liquids. Deficiency in surfactant protein D is a proven cause of respiratory failure in newborns, which is why some premature babies require supplementation with this protein. Previous studies, however, have not yielded favorable reports on the use of surfactant protein D in adults [7].

The BIS (Bispectral Index) is a method of neuromonitoring that utilizes EEG (Electroencephalography) to evaluate the brain’s electrical activity. Notably, ketamine, a pharmacological agent used for sedation, does not significantly influence BIS readings, which can pose a challenge in monitoring the depth of anesthesia when this drug is administered [5,8,9].

ARDS is one of the worst prognostic conditions for ICU admissions. The most common causes of this syndrome are circulatory failure, infections (pneumonia), and autoimmune factors [9]. However, cardiac issues are not believed to cause severe ARDS. ARDS is categorized according to the 2012 Berlin. ARDS is considered moderate when PaO_2_ is between 100 and 200 mmHg and severe when PaO_2_ is <100 mmHg. In adults with ARDS, the underlying cause is treated, i.e., in the case of pneumonia, antibiotic therapy is employed, and in the case of autoimmune diseases, steroid therapy is used, followed by targeted immunosuppressive medications [10]. Deep sedation in ARDS is particularly justified in the initial stages of treatment because reducing muscle oxygen consumption and suppressing the sympathetic nervous system leaves more oxygen available for critical organs [11]. Notably, extracorporeal therapies (CRRT and ECMO) and ARDS, often associated with infection, affect the concentration of midazolam [12,13,14].

In our study, we aimed to answer the following primary questions:

(1) Is there a correlation between RASS/BIS and midazolam dose/midazolam concentration?
(2) Do patients who die receive significant doses of other sedative drugs to maintain a similar state of sedation as other ARDS patients?

Our secondary questions were as follows:

(1) Do lung transplant recipients require significantly higher doses of sedative drugs to maintain a similar state of sedation as other ARDS patients?
(2) What are the concentrations of RAGE and surfactant protein D in ARDS patients?

## 2. Materials and Methods

A prospective, single-center investigation was carried out in the Intensive Care Unit of the academic hospital affiliated with the Medical University of Silesia in Katowice. Data collection took place between October 2022 and December 2024. The study population consisted of patients undergoing mechanical ventilation with an FiO_2_ exceeding 60%, as well as ventilated individuals requiring additional support such as ECMO (Extracorporeal Membrane Oxygenation), inhaled NO (Nitric Oxide) therapy, or ECCO2R (Extracorporeal Carbon Dioxide Removal) over 24 h before the study.

Participants under 18 years of age, those not receiving continuous midazolam infusion, or those who did not require mechanical ventilation were excluded. Ethical approval for the project was granted by the Bioethics Committee of the Medical University of Silesia in Katowice (PCN/022/KB1/55/II/21/22, dated 18 October 2022).

Randomization was not implemented, as the protocol did not involve testing a novel pharmacological agent or an innovative therapeutic intervention. The Ethics Committee of the Medical University of Silesia allowed the issuance of substitute consent from lineal family members (children, parents, or spouse). It was explained that this study was observational. Except for BIS and midazolam blood sampling, no other activities were planned. All patients were enrolled in the study by the first author.

All enrolled patients underwent twice-daily evaluations (at 9:00 and 21:00) to determine their level of sedation using the RASS (Richmond Agitation–Sedation Scale) scale, which ranges from −5 (no responsiveness) to +4 (severe agitation or delirium). Measurements obtained from RASS and BIS were then compared with serum midazolam concentrations. On each day, when blood samples for midazolam measurement were taken, additional laboratory tests assessing renal and hepatic function were also carried out. Kidney performance was estimated using the Cockcroft–Gault formula, while liver status was evaluated with the MELD (Model of End-Stage Liver Disease) score (ranging from 6.43 to 40 points). Subsequently, the relationship between midazolam levels and the occurrence of any complications was analyzed. The total drug dose administered during the previous 24 h (from 9:00 to 9:00) was recorded daily and compared with the corresponding serum concentrations. BIS monitoring was conducted using the COVIDIEN Medtronic system with dedicated BIS Quatro sensors. BIS assessment was performed on a scale of 0 to 100 points for some patients (only about 50% of RASS measurements have a corresponding BIS).

For individuals enrolled in the study, routine laboratory tests required for their clinical care were performed, and an additional EDTA blood sample (ethylenediaminetetraacetic acid, disodium salt) was collected twice weekly to determine midazolam concentrations. Sedation monitoring using the BIS device, along with RASS assessment, took place twice daily at 9:00 and 21:00. On the same days that midazolam samples were drawn, kidney and liver function tests, as well as lactate measurements, were also carried out. The analyses were performed via liquid chromatography–tandem mass spectrometry (LC-MS/MS) using the ClinMass^®^ TDM Kit System Benzodiazepines in Serum/Plasma (RECIPE Chemicals, Munich, Germany). Elimination was estimated based on the following formula: (dose × 1000)/(concentration × 24).

The measurement of RAGE and Human surfactant D protein was twice as high in the first and last instances of blood drawn with midazolam. The Human RAGE (MOK) ELISA Kit (ThermoFisher Scientific, Waltham, MA, USA) was used for RAGE testing. The tests consisted of administering 100 μL of diluted plasma samples, diluted 10-fold, on plates prepared according to the sandwich method. For surfactant protein D testing, the Human SP-D ELISA Kit (ThermoFisher Scientific, Waltham, MA, USA) was used. The testing consisted of administering 100 μL of diluted plasma samples, which were diluted 2-fold on plates prepared according to the sandwich method.

Elimination was estimated based on the following formula: (dose × 1000)/(concentration × 24).

As the effects of deep sedation subsided, polyneuropathy, delirium, and drug dependence were analyzed.

Continuous variables were described using the mean and standard deviation, whereas categorical data were expressed as proportions. Depending on the type of data, statistical differences between groups were examined using the chi-square test, the Mann–Whitney U test, Pearson’s correlation coefficient, or Spearman’s rank correlation.

The influence of selected independent factors on mortality was assessed through univariable logistic regression analysis. A *p*-value below 0.05 was considered indicative of statistical significance. All statistical computations and visualizations were carried out with the Dell Statistica software (version 13).

## 3. Results

In total, 25 patients were enrolled in this study. The mean cumulative dose of midazolam administered was 243.6 mg, and the average serum concentration reached 226 µg/L. Overall mortality was substantial, reaching 44% (11 patients). However, among the remaining individuals, 86% (12 patients) were alive at the six-month follow-up. Among the survivors, complications related to deep sedation occurred in 71% of cases. Detailed outcomes are summarized in Table 1.

All patients required additional sedative agents beyond midazolam. Propofol was used most frequently (96%), followed by ketamine or dexmedetomidine (84%). Opioids and thiopental were required by 80% and 60% of patients, respectively. The rarest was isoflurane administration, which was required by 8% of patients.

Patients who died had lower RASS scores than the others, with −3.8 ± 1.1 vs. −1 ± 1.6 points, *p* < 0.001. Additionally, these patients had higher APACHE II scores than the others, with 25.7 ± 5.1 vs. 19.4 ± 6.1 points, *p* = 0.01, and were older, 58 ± 9.5 vs. 41 ± 13.7, *p* = 0.004. We observed that the deceased received higher doses of midazolam compared to other patients to maintain the minimum level of required sedation (135.5 ± 75.1 mg vs. 39.6 ± 59.2 mg; *p* = 0.002), which yielded higher concentrations of both midazolam and α-OH-midazolam (148.6 ± 83.5 µg/L vs. 27.2 ± 36.1 µg/L; *p* < 0.001, and 18 ± 15.9 vs. 5.3 ± 6.1 µg/L; *p* < 0.001). The remaining results are provided in Table 1 and Table 2 and the Supplementary Tables S1–S4.

A negative correlation was observed between RASS and midazolam dosage (r = −0.44, *p* < 0.001, midazolam concentration: r = −0.33, *p* < 0.001) and α-OH-midazolam concentration (r = −0.24, *p* = 0.008). Similarly, a negative correlation was observed between BIS and midazolam concentration (r = −0.3, *p* = 0.016) and with α-OH-midazolam (r = −0.3, *p* = 0.016). However, we observed no correlation with midazolam dosage (r = −0.2, *p* = 0.118). There was also a positive correlation between lactate and MELD (Model of End-Stage Liver Disease) (r = 0.49, *p* < 0.001).

An additional analysis was performed to determine whether there was a difference in surfactant protein D and RAGE between the study population and the frozen material from the COVID-19+ population. We found that patients with COVID-19 had a higher surfactant protein D concentration but no difference in RAGE (4.8 ± 7.6 vs. 8.6 ± 8.4 ng/mL; *p* = 0.021 and 262 ± 690 vs. 111.6 ± 138.3 pg/mL; *p* = 0.677). RAGE correlated positively with inflammatory factors such as IL-6 (r = 0.93, *p* < 0.001), PCT (procalcitonin) (r = 0.69, *p* < 0.001), and CRP (C-Reactive Protein) (r = 0.46, *p* = 0.005). Surfactant protein D correlated positively with tidal volume (r = 0.53, *p* = 0.001).

Candidates for lung transplantation demonstrated significantly lower lung volume than that of other patients (212 ± 127 mL versus 527 ± 201 mL; *p* < 0.001). The maximum α-OH-midazolam serum levels were also higher in this group (139 ± 116.6 µg/L compared with 64.1 ± 70 µg/L in the non-transplant group; *p* = 0.035). These individuals experienced longer ICU stays, averaging 46 ± 23 days versus 37 ± 18 days (*p* = 0.005), and required prolonged tracheostomy ventilation of 38 ± 20 vs. 15 ± 15 days (*p* < 0.001). Additional results are provided in the Supplemental Materials.

Patients who underwent lung transplantation had a worse pO_2_/FiO_2_ ratio and tidal volume compared with those in the remaining group (132.4 ± 76.7 vs. 169.1 ± 80.3 mmHg; *p* = 0.03 and 252.4 ± 108.1 vs. 480 ± 214.3 ml; *p* < 0.001).

## 4. Discussion

Canadian researchers suggest that routine monitoring of midazolam concentrations is unnecessary for all individuals receiving this medication [2].

Neurological complications occurred in 71.4% of the 14 patients who survived. The majority had polyneuropathy, while some presented psychosis and dependence. However, sedation is not the sole cause of these complications; other causes include multiple organ failure, immobilization, use of other medications, and prolonged ventilation. Furthermore, continuous midazolam infusion is significantly more likely to increase drug tolerance [15]. On the other hand, the sedation of patients with ARDS is not performed with multiple drugs, including neuromuscular blocking drugs [10].

In this study, the survival rate was 56%, which is within the norm described in other reports; survival in severe ARDS was reported to be 50–60% [7].

Six-month survival was good at 86%. Of the 14 patients who survived the ICU stay, only two died within 6 months. The available literature shows similar results, with mortality in ARDS patients occurring primarily during the acute phase and decreasing thereafter, while six-month mortality ranges from 5% to 80% [16,17,18]. Naturally, these patients require intensive rehabilitation and care, as they are in poorer mental and physical condition. Prolonged sedation and immobilization, moreover, worsen muscle function [18,19,20].

A German study reported no association between BIS measurements, the RASS, and drug concentrations in the bloodstream. However, a 2004 investigation by Bremer, cited by the German authors and based on a substantially larger patient cohort, demonstrated a statistically significant relationship [1].

Given the potential adverse effects of sedative agents, including dependence and tolerance, accurately assessing sedation depth remains essential. Both BIS monitoring and the RASS scale serve as valuable tools, allowing clinicians to evaluate sedation quality and detect signs of patient awakening during routine nursing activities [8,9,21].

Higher patient mortality was observed in patients with a higher burden of disease and at older ages, which has been uniformly reported in previous ICU publications [22]. These patients also had higher lactate concentrations compared to the rest of the population. Furthermore, a positive correlation between lactate and MELD was demonstrated. Notably, lactate is a recognized marker of decompensated shock [23].

Due to the lung transplant program at the study hospital, some patients with severe lung failure were transferred to the ICU from other ICUs for lung transplant qualification. These patients were in better condition than the remaining population, as evidenced by their lower lactate and IL-6 levels. Additionally, these patients already had features of chronic respiratory failure, such as longer ventilation time via tracheostomy, lower tidal volume, and hypercapnia. Ultimately, four patients received transplantation. Both ventilator therapy and ECMO are bridges to transplantation, but these patients benefited less from lung transplantation than those who did not require ICU beforehand [24,25,26].

Extracorporeal therapies alter the pharmacokinetics of midazolam by increasing the volume of distribution. ECMO, in particular, has a predisposition to alter the pharmacokinetics of lipophilic drugs [13,14]. There are reports that CRRT does not affect blood midazolam concentrations [12]. Moreover, pulmonary patients often suffer cardiological complications. The situation was similar in our study population, as the hospital is primarily a cardiology hospital. There are reports that pulmonary edema may have features of ARDS, which requires the implementation of NIV or HFNOT. ECMO A-V, especially in the fem–fem configuration, may cause pulmonary edema [27,28].

Preliminary biomarker testing was performed in high-risk patients. Due to the small sample size, however, further studies are needed in larger groups of patients. A potential marker of ARDS is surfactant protein D, which was not found to differ significantly in this study. However, this protein is a good marker, especially in children: The higher the serum concentrations, the greater the risk of severe ARDS [29]. An additional analysis showed that patients with severe COVID-19 from the pilot study had higher surfactant protein D concentrations than those in the main population. In a published meta-analysis from 2024, Iranian researchers reported that elevated surfactant protein D concentrations positively correlated with the severity of COVID-19 [30]. Another potential marker is RAGE. In a previous study, the depletion of RAGE in knockout mice caused lung fibrosis [30]. Another publication, however, describes a completely different mechanism: Mice lacking RAGE were protected from pulmonary fibrosis and severe septic shock [6]. Soluble RAGE (sRAGE), however, has the opposite effect, acting instead as an anti-inflammatory. Patients had lower RAGE levels during V-V ECMO therapy, and a similar situation was observed in the population of potential lung transplant candidates. This result is likely due to the fact that some patients on ECMO, and most of those awaiting lung transplantation, had lung fibrosis, which reduced RAGE levels in these patients. RAGE activates signaling to activate proinflammatory genes [6,31,32,33].

One limitation of the present study is its single-center nature. Our study also has significant methodological limitations due to the small sample size and data structure. Numerous repeated measurements of clinical and pharmacokinetic parameters (e.g., RASS, BIS, PaO_2_/FiO_2_, midazolam dose, and midazolam concentration) were performed in many patients. Due to the small number of patients, advanced methods for analyzing repeated data, such as mixed models or GEE, which require a larger sample size to obtain stable estimates, were not used.

To reduce the risk of artificially inflating statistical significance, the data were ultimately analyzed at the patient level by aggregating measurements (e.g., mean or median values for each patient). Nevertheless, the results should be considered exploratory, and the observed relationships—particularly between midazolam dose and concentration and clinical outcomes—cannot be interpreted in terms of causality. Larger, prospective studies using full models for repeated data are necessary. Due to the limited sample size, multivariate analysis was not performed. The results should thus be interpreted as observational and potentially confounded by disease severity. Therefore, we cannot conclude a causal relationship. This study was designed in 2020/2021, during the peak of the COVID-19 pandemic in Poland, and was initially focused on this pathology. At that time, one section of the ICU was dedicated exclusively to COVID-19 treatment. After obtaining a grant and purchasing materials, COVID-19 cases decreased significantly, and the number of patients requiring ICU stay decreased even further. Thus, this observational study primarily generated hypotheses for future ARDS studies. Due to the nature of the pandemic, the number of patients enrolled was significantly lower than planned. Another drawback is that not all patients underwent bispectral analysis due to the irreversible failure of previous equipment and the need to purchase new equipment. Midazolam monitoring was also impaired by the effects of other drugs. An advantage of this study, however, is that all patients were enrolled by the first author, which reduced subjectivity in patient selection, as well as the accuracy of each observation and its prospective nature.

## 5. Conclusions

Overall, routine monitoring of midazolam was not found to provide additional clinical value. Further studies, however, are needed in high-risk groups. Moreover, prolonged sedation causes neurological complications. Despite the high mortality rate in the ICU for patients with severe respiratory failure, the six-month survival rate for discharged patients remained high, reaching over 80%.

## Figures and Tables

**Table 1:** Demographic parameters and comparison between deceased patients and patients who survived.

	*N*		*n*			%	
death	25		11			44.0%	
women	25		9			36.0%	
6 months survival	14		12			85.7%	
NIV	25		12			48.0%	
tracheostomy	25		17			68.0%	
acute lung failure	25		19			76.0%	
kidney injury	25		20			80.0%	
liver failure	25		12			48.0%	
cardiac failure	25		19			76.0%	
pulmonary hypertension	21		11			52.4%	
CVVHD/HDF	25		11			44.0%	
ECCO2R	25		5			20.0%	
NO	25		5			20.0%	
prone position	25		14			56.0%	
COVID-19	25		2			8.0%	
ECMO V-V	25		14			56.0%	
catecholamines	25		23			92.0%	
	*N*		mean			SD	
APACHE II (points)	25		22.160			6.459	
SAPS III (points)	25		56.720			13.139	
age (years)	25		48.560			14.540	
ICU stay (days)	25		33.560			21.645	
ventilator (days)	25		30.080			19.399	
weight (kg)	25		90.000			25.252	
LVEF (%)	22		47.000			8.954	
	death			alive			*p*
	*N*	*n*	%	*N*	*n*	%	Yates
women	11	4	36.4%	14	5	35.7%	0.699
NIV	11	3	27.3%	14	9	64.3%	0.151
tracheostomy	11	6	54.5%	14	11	78.6%	0.397
acute lung failure	11	9	81.8%	14	10	71.4%	0.895
kidney injury	11	10	90.9%	14	10	71.4%	0.481
liver failure	11	7	63.6%	14	5	35.7%	0.325
cardiac failure	11	9	81.8%	14	10	71.4%	0.895
Child–A (points)	11	4	36.4%	14	13	92.9%	**0.010**
Child–B (points)	11	7	63.6%	14	1	7.1%	**0.010**
CVVHD/HDF (general ICU stay)	11	9	81.8%	14	7	50.0%	0.220
CVVHD/HDF (research time)	11	6	54.5%	14	5	35.7%	0.592
plasmapheresis	11	1	9.1%	14	1	7.1%	0.573
SPAD	11	3	27.3%	14	0	0.0%	0.143
Cytosorb	11	1	9.1%	14	1	7.1%	0.573
ECCO2R	11	1	9.1%	14	4	28.6%	0.481
NO	11	2	18.2%	14	3	21.4%	0.763
vasopressor	11	10	90.9%	14	13	92.9%	0.573
neuromuscular blockade	11	7	63.6%	14	11	78.6%	0.706
prone position	11	6	54.5%	14	8	57.1%	0.783

NIV—Non-Invasive Ventilation; CVVHD/HDF—Continuous Venovenous Hemodialysis/Hemodiafiltration; ECCO2R—Extracorporeal Carbon Dioxide Removal; ECMO—Extracorporeal Membrane Oxygenation; NO—Nitric Oxide; APACHE II—Acute Physiology and Chronic Health Evaluation II; SAPS III—The Simplified Acute Physiology Score III; ICU—Intensive Care Unit; LVEF—Left Ventricular Ejection Fraction.

**Table 2:** Comparison between deceased patients and patients who survived.

	Deceased			Alive			*p*
	*n*	mean	SD	*n*	mean	SD	U M-W
APACHE II (points)	11	25.7	5.1	14	19.4	6.1	**0.010**
SAPS III (points)	11	58.5	13.2	14	55.3	13.4	0.529
age (years)	11	58.0	9.5	14	41.1	13.7	**0.004**
ICU stay (days)	11	27.7	21.9	14	38.1	21.1	0.162
ventilator (days)	11	27.1	21.7	14	32.4	17.8	0.311
midazolam dose—min (mg)	11	135.5	75.1	14	39.6	59.2	**0.002**
midazolam dose—max (mg)	11	255.0	121.3	14	390.7	160.3	0.052
midazolam dose/weight—min (mg/kg)	11	1.6	1.1	14	0.5	0.7	**0.007**
midazolam dose/weight—max (mg/kg)	11	2.9	1.6	14	4.5	1.8	0.059
midaz. serum concentration—min (µg/L)	11	148.6	83.5	14	27.2	36.1	**<0.001**
midaz. serum concentration—max (µg/L)	11	414.3	225.1	14	450.6	259.1	0.805
α-OH-midaz. ser. conc.—min (µg/L)	11	18.0	15.9	14	5.3	6.1	**0.001**
α-OH-midaz. ser. conc.—max (µg/L)	11	92.7	120.0	14	100.5	83.0	0.494

APACHE II—Acute Physiology and Chronic Health Evaluation II; SAPS III—The Simplified Acute Physiology Score III; ICU—Intensive Care Unit; midaz.—midazolam; α-OH-midaz. ser. conc.—α-hydroxymidazolam serum concentration.

## Data Availability

Data are contained within the article.

## Supplementary Materials

The following supporting information can be downloaded at: https://www.mdpi.com/article/10.3390/medicina62040742/s1, Table S1. Comparison between patient undergoing lung transplantation and not undergoing lung transplantation (per patients patients). Table S2. Comparison between patients undergoing lung transplantation and not undergoing lung transplantation (per measure variables). Table S3. Comparison between patients undergoing lung transplantation and not undergoing lung transplantation (per patients patients). Table S4. Comparison between patients who died versus alive.

## Abbreviations

The following abbreviations are used in this manuscript:

ICU	Intensive Care Unit
GABA	Gamma-aminobutyric Acid
ARDS	Acute Respiratory Distress Syndrome
RAGE	Receptor for Advanced Glycation Endproducts
AGEs	Advanced Glycation Endproducts
EEG	Electroencephalography
RASS	Richmond Agitation–Sedation Scale
NIV	Non-Invasive Ventilation
CVVHD	Continuous Venovenous Hemodialysis
CVVHDF	Continuous Venovenous Hemodiafiltration
SPAD	Single-Pass Albumin Dialysis
ECCO2R	Extracorporeal Carbon Dioxide Removal
ECMO	Extracorporeal Membrane Oxygenation
NO	Nitric Oxide
APACHE II	Acute Physiology and Chronic Health Evaluation II
SAPS III	The Simplified Acute Physiology Score III
LVEF	Left Ventricular Ejection Fraction
MELD	Model of End-Stage Liver Disease
BIS	Bispectral Index
IL-6	Interleukin 6
CRP	C-Reactive Protein

## References

[1] Nies R.J., Müller C., Pfister R., Binder P.S., Nosseir N., Nettersheim F.S., Kuhr K., Wiesen M.H., Kochanek M., Michels G. (2018). Monitoring of sedation depth in intensive care unit by therapeutic drug monitoring? A prospective observation study of medical intensive care patients. J. Intensive Care.

[2] Spina S.P., Ensom M.H. (2007). Clinical Pharmacokinetic Monitoring of Midazolam in Critically Ill Patients. Pharmacotherapy.

[3] Fragen R.J. (1997). Pharmacokinetics and Pharmacodynamics of Midazolam Given Via Continuous Intravenous Infusion in Intensive Care Units. Clin. Ther..

[4] Aoyama T., Hirata K., Yamamoto Y., Yokota H., Hayashi H., Aoyama Y., Matsumoto Y. (2016). Semi-mechanistic autoinduction model of midazolam in critically ill patients: Population pharmacokinetic analysis. J. Clin. Pharm. Ther..

[5] Tripathi M., Kumar V., Kalashetty M.B., Malviya D., Bais P.S., Sanjeev O.P. (2017). Comparison of Dexmedetomidine and Midazolam for Sedation in Mechanically Ventilated Patients Guided by Bispectral Index and Sedation-Agitation Scale. Anesth. Essays Res..

[6] Hergrueter A.H., Nguyen K., Owen C.A. (2011). Matrix metalloproteinases: All the RAGE in the acute respiratory distress syndrome. Am. J. Physiol. Lung Cell Mol. Physiol..

[7] Dushianthan A., Grocott M.P.W., Murugan G.S., Wilkinson T.M.A., Postle A.D. (2023). Pulmonary Surfactant in Adult ARDS: Current Perspectives and Future Directions. Diagnostics.

[8] Prottengeier J., Moritz A., Heinrich S., Gall C., Schmidt J. (2014). Sedation assessment in a mobile intensive care unit: A prospective pilot-study on the relation of clinical sedation scales and the bispectral index. Crit. Care.

[9] Sackey P.V., Radell P.J., Granath F., Martling C.R. (2007). Bispectral index as a predictor of sedation depth during isoflurane or midazolam sedation in ICU patients. Anaesth. Intensive Care.

[10] Lehingue S., Hraiech S., Papazian L., Chiumello D., Maciejewski D. (2021). Pharmacological Interventions: Neuromuscular Blocking Agents. Acute Respiratory Distress Syndrome.

[11] Chanques G., Constantin J.-M., Devlin J.W., Ely E.W., Fraser G.L., Gelinas C., Girard T.G., Guerin C., Jabaudon M., Jaber S. (2020). Analgesia and sedation in patients with ARDS. Intensive Care Med..

[12] Smeets T.J., de Geus H.R., Valkenburg A.J., Baidjoe L., Gommers D.A., Koch B.C., Hunfeld N.G., Endeman H. (2024). The Clearance of Midazolam and Metabolites during Continuous Renal Replacement Therapy in Critically Ill Patients with COVID-19. Blood Purif..

[13] Bertin S., Haefliger D., Schneider A.G., Giraud R., Perez M.-H., Bechtold X., Di Paolo E.R., Rothuizen L.E., Buclin T., Livio F. (2025). Effects of Extracorporeal Membrane Oxygenation Circuits on Drug Sequestration: A Review of Ex Vivo Experiments. J. Clin. Med..

[14] Kim H., Jin B.H., Yang S., Hahn J., Kang S., Kim D., Lee H., Kwack H., Chae S.U., Bae S.K. (2026). Effect of Extracorporeal Membrane Oxygenation Flow Rate on Midazolam Clearance: A Population Pharmacokinetic Study. Anesthesiology.

[15] Midazolam Material Safety Data Sheet. https://rejestry.ezdrowie.gov.pl/api/rpl/medicinal-products/8752/characteristic.

[16] Lopez Saubidet I., Maskin L.P., Rodríguez P.O., Bonelli I., Setten M., Valentini R. (2016). Mortality in patients with respiratory distress Syndrome. Med. Intensiv..

[17] Schmidt M., Zogheib E., Rozé H., Repesse X., Lebreton G., Luyt C.-E., Trouillet J.-L., Brechot N., Nieszkowska A., Dupont H. (2013). The PRESERVE mortality risk score and analysis of long-term outcomes after extracorporeal membrane oxygenation for severe acute respiratory distress syndrome. Intensive Care Med..

[18] Fazzini B., Battaglini D., Carenzo L., Pelosi P., Cecconi M., Puthucheary Z. (2022). Physical and psychological impairment in survivors of acute respiratory distress syndrome: A systematic review and metaanalysis. Br. J. Anaesth..

[19] Thomas R., Turaka V.P., Peter J.V., Christopher D.J., Balamugesh T., Mahasampath G., Mathuram A.J., Sadiq M., Ramya I., George T. (2022). Good survival rate, moderate overall and good respirator quality of life, near normal pulmonary functions, and good return to work despite catastrophic economic costs 6 months following recovery from Acute Respiratory Distress Syndrome. Lung India.

[20] Bein T., Weber-Carstens S., Apfelbacher C. (2018). Long-term outcome after the acute respiratory distress syndrome: Different from general critical illness?. Curr. Opin. Crit. Care.

[21] Zheng J., Gao Y., Xu X., Kang K., Liu H., Wang H., Yu K. (2018). Correlation of bispectral index and Richmond agitation sedation scale for evaluating sedation depth: A retrospective study. J. Thorac. Dis..

[22] Pasieka P., Surówka A., Fronczek J., Skwara E., Czuczwar M., Borys M., Krawczyk P., Ziętkiewicz M., Nowak Ł.R., Żukowski M. (2024). Prevalence of life-sustaining treatment limitations in Polish very old intensive care patients (VIPs). A post-hoc analysis of two prospective observational studies. J. Crit. Care.

[23] Kutnik P., Szczukocka M., Borys M., Czuczwar M. (2019). Procalcitonin dynamics, lactates, and haemoglobin serum levels might be a useful predictive tool of mortality in patients undergoing veno-venous extracorporeal oxygenation membrane support. Single centre experience. Anaesthesiol. Intensive Ther..

[24] Lee S.Y., Ahn J.H., Kim H.C., Shim T.S., Kang P.-J., Lee G.D., Choi S.H., Jung S.-H., Park S.-I., Hong S.-B. (2024). Outcomes of Lung Transplantation in Patients with Right Ventricular Dysfunction: A Single-Center Retrospective Analysis Comparing ECMO Configurations in a Bridge-to-Transplant Setting. Transpl. Int..

[25] Faccioli E., Terzi S., Pangoni A., Lomangino I., Rossi S., Lloret A., Cannone G., Marino C., Catelli C., Dell’Amore A. (2021). Extracorporeal membrane oxygenation in lung transplantation: Indications, techniques and results. World J. Transplant..

[26] Dellavolpe J., Czuczwar M. (2024). ECMO.

[27] Tetaj N., Capecchi G., Rubino D., Stazi G.V., Cingolani E., Lesci A., Segreti A., Grigioni F., Bocci M.G. (2026). Respiratory Support in Cardiogenic Pulmonary Edema: Clinical Insights from Cardiology and Intensive Care. J. Cardiovasc. Dev. Dis..

[28] Tetaj N., Nusca A., Piccirillo F., Halasz G., Gabrielli D., Ussia G.P., Grigioni F. (2026). Short-Term Percutaneous Mechanical Circulatory Support in Acute Coronary Syndrome with Cardiogenic Shock: Which Device to Choose?. J. Cardiovasc. Dev. Dis..

[29] Dahmer M.K., Flori H., Sapru A., Kohne J., Weeks H.M., Curley M.A., Matthay M.A., Quasney M.W. (2020). Surfactant Protein D Is Associated with Severe Pediatric ARDS, Prolonged Ventilation, and Death in Children with Acute Respiratory Failure. CHEST.

[30] Bastani M.N., Jalilian S., Bahreiny S.S., Makvandi M., Aghaei M., Mansouri Z., Karamali N., Sakhavarz T., Amraei M., Harooni E. (2024). Update prognostic potency of surfactant protein D (SP-D) in the COVID-19 landscape: An In-depth meta-analytical exploration. Biomark. Med..

[31] Yamaguchi K., Iwamoto H., Sakamoto S., Horimasu Y., Masuda T., Miyamoto S., Nakashima T., Fujitaka K., Hamada H., Hattori N. (2022). Association of the RAGE/RAGE-ligand axis with interstitial lung disease and its acute exacerbation. Respir. Investig..

[32] Johnson L.L., Tekabe Y., Zelonina T., Ma X., Zhang G., Goldklang M., D’Armiento J. (2023). Blocking RAGE expression after injury reduces inflammation in mouse model of acute lung injury. Respir. Res..

[33] Salehi M., Amiri S., Ilghari D., Hasham L.F., Piri H. (2023). The Remarkable Roles of the Receptor for Advanced Glycation End Products (RAGE) and Its Soluble Isoforms in COVID-19: The Importance of RAGE Pathway in the Lung Injuries. Indian J. Clin. Biochem..

