# Supplementary material for: Drug Monitoring of Therapy with Midazolam in Patients with ARDS: A Single-Center Prospective Study
Source: Medicina (Kaunas). 2026 Apr 13;62(4):742. doi: 10.3390/medicina62040742 (PMC13117684; doi:10.3390/medicina62040742)
Supplement: Supplementary file 1 [file medicina-62-00742-s001.zip › medicina-4228535-supplementary.pdf]

Supl. Table S1. Comparison between patients undergoing lung transplantation and those not undergoing lung transplantation (per patient).

	patients undergoing lung transplantation evaluation			patients not undergoing lung transplantation evaluation			p
	n	mean	SD	n	mean	SD	U M-W
APACHE II (points)	11	23.1	5.6	14	21.4	7.2	0.805
SAPS III (points)	11	58.6	12.9	14	55.2	13.6	0.493
age (years)	11	46.2	14.5	14	50.4	14.8	0.380
ICU stay (days)	11	36.6	23.6	14	31.1	20.5	0.547
ventilator (days)	11	33.7	23.8	14	27.2	15.5	0.722
intubation time (days)	11	10.7	11.2	14	13.8	7.3	0.217
tracheostomy + ventliator (days)	10	27.9	18.9	13	12.1	15.4	**0.024**
midazolam dose/weight – min (mg/kg)	11	1.4	1.3	14	0.6	0.7	0.164
midazolam dose/weight – max (mg/kg)	11	4.8	1.9	14	3.0	1.4	**0.019**
α-OH-midaz.ser.conc.-min(µg/L	11	13.5	17.3	14	8.8	8.3	0.642
α-OH-midaz. ser. conc. - max (µg/L)	11	139.0	116.6	14	64.1	69.9	**0.035**

APACHE II—Acute Physiology and Chronic Health Evaluation II, SAPS III—The Simplified Acute Physiology Score III, ICU—Intensive Care Unit, midaz.—midazolam, and α-OH-midaz. ser. conc.—α-hydroxymidazolam serum concentration.

Supl. Table S2. Comparison between patients undergoing lung transplantation and those not undergoing lung transplantation (per measure variables).

	patients undergoing lung transplantation evaluation			patients not undergoing lung transplantation evaluation			p
	n	mean	SD	n	mean	SD	U M-W
RASS (points)	82	-3.7	1.4	66	-3.2	1.9	0.086
BIS (points)	55	53.7	17.0	28	59.2	18.2	0.257
pO2/FiO2 (mmHg)	70	130.3	74.6	71	147.7	73.9	0.054
midazolam dose (mg)	72	253.8	146.5	71	233.4	144.7	0.438
midazolam dose/weight (mg/kg)	72	3.4	1.9	71	2.3	1.4	**0.001**
midaz. serum concentration (µg/L)	73	239.9	204.0	72	211.8	148.8	0.729
tidal volume (ml)	57	211.7	127.1	44	527.1	201.3	**<0.001**
NT-proBNP (pg/ml)	28	8875.6	13293.3	34	1181.6	2972.7	**<0.001**
lactate (mmol/l)	70	1.0	0.5	71	1.5	1.4	**<0.001**
pO2 (mmHg)	71	79.5	18.6	71	85.9	19.2	0.055
pCO2 (mmHg)	71	44.7	6.2	71	42.2	10.6	**0.001**
procalcitonin (ng/ml)	43	3.9	11.7	48	1.9	2.9	0.427
IL-6 (pg/ml)	39	22.1	122.2	44	409.1	1864.9	**<0.001**

RASS—Richmond Agitation–Sedation Scale, BIS—Bispectral Index, midaz.—midazolam, α-OH-midaz. ser. conc.—α-hydroxymidazolam serum concentration, and IL-6—Interleukin 6

Supl. Table S3. Comparison between patients undergoing lung transplantation and those not undergoing lung transplantation (per patient).

	patients undergoing lung transplantation evaluation			patients not undergoing lung transplantation evaluation			p
	N	n	%	N	n	%	Yates
death	11	5	45.5%	14	6	42.9%	0.783
women	11	5	45.5%	14	4	28.6%	0.650
6 months survival	6	4	66.7%	8	8	100.0%	0.321
tracheostomy	11	9	81.8%	14	8	57.1%	0.378
acute lung failure	11	6	54.5%	14	13	92.9%	0.079
cardiac failure	11	9	81.8%	14	10	71.4%	0.895
pulmonary hypertension	11	7	63.6%	10	4	40.0%	0.518
CVVHD/HDF	11	7	63.6%	14	4	28.6%	0.178
ECCO2R	11	4	36.4%	14	1	7.1%	0.190
NO	11	1	9.1%	14	4	28.6%	0.481
prone position	11	4	36.4%	14	10	71.4%	0.178
COVID-19	11	0	0.0%	14	2	14.3%	0.573
ECMO V-V	11	9	81.8%	14	5	35.7%	0.058

CVVHD/HDF—Continuous Venovenous Hemodialysis/Hemodiafiltration, ECCO2R—Extracorporeal Carbon Dioxide Removal, and ECMO—Extracorporeal Membrane Oxygenation

Supl. Table S4. Comparison between patients who died versus alive patients.

	death			alive			p
	n	mean	SD	n	mean	SD	U M-W
RASS (points)	47	-4.2	1.0	101	-3.2	1.8	**<0.001**
BIS (points)	32	56.8	18.4	51	54.8	17.0	0.660
pO2/FiO2 (mmHg)	52	103.9	45.2	89	159.6	80.6	**<0.001**
midazolam dose/weight (mg/kg)	54	2.7	1.3	89	3.0	2.0	0.450
elimination (L/24)	54	45.8	23.2	77	119.4	306.7	**<0.001**
tidal volume (ml)	39	251.9	211.2	62	410.3	215.1	**<0.001**
total bilirubin (µmol/l)	45	32.8	66.0	65	13.0	8.3	**0.009**
INR	46	1.4	0.4	74	1.3	0.2	0.101
MELD (points)	46	12.0	4.8	75	10.2	2.7	0.081
lactate (mmol/l)	53	1.6	1.7	88	1.1	0.5	**0.010**
pO2 (mmHg)	53	77.8	21.2	89	85.6	17.2	**0.010**
pCO2 (mmHg)	53	45.5	9.8	89	42.2	7.8	**0.034**
procalcitonin (ng/ml)	38	2.1	3.3	53	3.4	10.5	0.557
IL-6 (pg/ml)	31	472.7	2176.5	52	80.9	398.7	0.760
CRP (mg/l)	35	179.8	115.2	57	126.5	88.0	**0.032**

RASS—Richmond Agitation–Sedation Scale, BIS—Bispectral Index, MELD—Model of End-Stage Liver Disease, IL-6—interleukin 6, and CRP—C-Reactive Protein
